# Role of DJ-1 in Immune and Inflammatory Diseases

**DOI:** 10.3389/fimmu.2020.00994

**Published:** 2020-06-16

**Authors:** Lulu Zhang, Jincheng Wang, Jiajia Wang, Bo Yang, Qiaojun He, Qinjie Weng

**Affiliations:** Center for Drug Safety Evaluation and Research, College of Pharmaceutical Sciences, Zhejiang University, Hangzhou, China

**Keywords:** DJ-1, ROS, immune diseases, inflammatory diseases, therapeutic target

## Abstract

The DJ-1 protein, known as an oxidative stress sensor, participates in the onset of oxidative stress-related diseases such as cancer, neurodegenerative disorders, type 2 diabetes, and male infertility. Although DJ-1 has been extensively studied for more than two decades, evidence has only recently emerged that it plays a key role in immune and inflammatory disorders. The immune regulatory function of DJ-1 is achieved by modulating the activation of several immune cells including macrophages, mast cells, and T cells via reactive oxygen species (ROS)-dependent and/or ROS-independent mechanisms. This review describes the current knowledge on DJ-1, focusing on its immune and inflammatory regulatory roles, and highlights the significance of DJ-1 as a novel therapeutic target for immune and inflammatory diseases.

## Introduction

The *DJ-1* gene was originally discovered as an oncogene that shows transforming activity in conjunction with the *ras* gene and was later identified as a causative gene for autosomal recessive, early-onset Parkinson's disease (PD), *PARK7* (1, 2). As a multifunctional protein, DJ-1 can regulate transcription and signal transduction pathways, scavenge reactive oxygen species (ROS), and function as a molecular chaperone and enzyme, all of which leads to the anti-oxidative stress reaction of DJ-1 (3–7). In addition to the participation of DJ-1 in cancer and PD, a great number of studies have indicated that DJ-1 is also involved in the pathogenesis of multiple oxidative stress-associated diseases including stroke, male infertility, neurodegenerative diseases, and diabetes mellitus (8–11).

Recently, accumulating evidence has indicated that DJ-1 exerts immune and inflammatory regulatory functions by modulating the activation of several immune cells such as macrophages, mast cells (MCs), and T cells via ROS-dependent and/or ROS-independent mechanisms (12–14). In this review, we discuss the role of DJ-1 in the physiopathology of several immune and inflammatory diseases including sepsis, allergic diseases, atherosclerosis (AS), and multiple sclerosis (MS), highlighting DJ-1 as a potential therapeutic target for immune and inflammatory diseases.

## Gene, Protein, and Molecular Characteristics of DJ-1

The human *DJ-1* gene (*PARK7*) maps on chromosome 1 at 1p36.23 according to Entrez Gene, and the mouse homolog is found on chromosome 4E. Containing 17 distinct gt-ag introns and 7 exons, *DJ-1* encompasses 23.86 kb (15). Through alternative promoters and alternative splicing, 17 different variants from the gene have been reported (15). Among these, 15 transcripts have protein-coding potential, while 2 transcript variants encoding the same protein have been identified for this gene (15).

Comprising 189 amino acid residues, human DJ-1 is a small (20 kDa) protein that belongs to the large DJ-1/ThiJ/PfpI superfamily, which is ubiquitously expressed in more than 22 human tissues including the pancreas, kidney, skeletal muscle, liver, testis, and heart (16). During the course of many years, the DJ-1/ThiJ/PfpI superfamily has been found in diverse organisms from bacteria to humans (16). Although not all members of this superfamily have been characterized structurally, 61 structures of human DJ-1 that were determined by X-ray crystallography have been deposited in the Protein Data Bank as of January 2020, indicating that it exists as a dimer (17). The structure of the DJ-1 monomer has a helix-fold-helix flavodoxin-like fold, with 11 β-strands (β1–β11) and 8 α-helices (αA–αH) (18). It centers on a β-sheet that contains six parallel strands arranged regularly and surrounded by α-helices, and β3–4 that form a hairpin structure that contributes to its dimerization (17–19). In addition, dimeric DJ-1 characterizes a different arrangement of the αA and αH helices of the two monomers from other members of this superfamily (17). However, the L166P (Leu^166^Pro) mutation of DJ-1, which is located in the center of αG and linked to familial PD, confers reduced protein stability and interferes with homodimerization (20).

DJ-1 seems to act primarily as a redox-sensitive chaperone and sensor for imbalanced cellular redox state, since its overexpression is induced by a variety of oxidative agents such as paraquat, lipopolysaccharide (LPS), iron, hydrogen peroxide (H_2_O_2_), 6-hydroxydopamine, ultraviolet irradiation, and high glucose, protecting multiple kinds of cells including endothelial cells, macrophages, fibroblast cells, neurons, cancer cells, and islet β cells (11, 21–25). Deletion of *DJ-1* augments cell death by oxidative stress, endoplasmic reticulum stress, and proteasome inhibitors (6).

## The Multifaceted Roles of DJ-1 as an Antioxidant

### Reduced and Oxidized Forms of DJ-1 and Their Functions

DJ-1 functions as an antioxidant through various mechanisms, including scavenging ROS in a manner dependent on three redox-sensitive cysteine residues at amino acids 46, 53, and 106 (C46, C53, and C106, respectively). Of these, C106 is considered the most oxidative stress-sensitive residue and is sequentially oxidized to form sulfenic acid (SOH), sulfinic acid (SO_2_H), and sulfonic acid (SO_3_H) (5). DJ-1 exhibits distinct properties and functions dependent on the oxidation state of C106. Moderate oxidation of C106 to SO_2_H is responsible for mitochondrial localization of DJ-1 and inhibits fibrillation of α-synuclein (26–28). Additionally, the cytoprotective interaction of DJ-1 with apoptosis signal-regulating kinase 1 (ASK1) was mediated by SO_2_H and modulated by peripheral C46 and C53 (29, 30). However, high oxidation of C106 to SO_3_H results in aggregated and inactive DJ-1, and has been correlated with the increased progression of disease including PD where oxidative stress is a part of the pathophysiology (27, 31).

The reduced form of DJ-1 exerts various functions in addition to eliminating excessive ROS. In terms of DJ-1-dependent activation of dopamine biosynthesis by two enzymes, tyrosine hydroxylase and levodopa decarboxylase, DJ-1 possessing reduced and SOH forms of C106 is active and binds to two enzymes, positively regulating their activities (32). Moreover, the reduced form of DJ-1 is required for interaction with phosphatase and tensin homolog and inhibits its phosphatase activity in NIH3T3 fibroblasts, which is not sustained with prolonged oxidative stress and highly oxidized forms of DJ-1 (33). A recent study reported that the direct binding between reduced DJ-1 and Lyn kinase is an indispensable step for full Lyn activation and IgE-mediated stimulation in human MCs, indicating that MC signaling is largely unrelated to DJ-1 antioxidant activity (34).

### DJ-1 as a Regulator of Mitochondrial Homeostasis

Under physiological conditions, DJ-1 is predominantly present in the cytoplasm and, to a lesser extent, in the nucleus and mitochondria including the outer membrane, matrix, and intermembrane space of mitochondria (26, 35). However, upon oxidative stress, cytoplasmic DJ-1 translocates to the mitochondria and subsequently to the nucleus, while mitochondrial localized DJ-1 exhibits stronger cytoprotective effects against oxidative stress than cytosolic or nuclear DJ-1 (36). The capability of immediate redistribution of DJ-1 according to changes in the microenvironment is crucial for regulating mitochondrial homeostasis and function, coinciding with cytoprotective activity (26, 36, 37). Studies have shown that loss of DJ-1 leads to mitochondrial dysfunction including decreased respiratory control ratio, mitochondrial membrane potential, ATP levels, and impaired dynamics, *in vitro* and *in vivo* (37–40).

The mitochondrial translocation of DJ-1 is likely mediated by chaperones in response to oxidative stress (41). DJ-1 colocalizes with heat shock protein 70 (Hsp70) in the cytoplasm and is associated with mtHsp70/Grp75, a mitochondria-resident Hsp70 (41). Therefore, DJ-1 homodimers translocate to the mitochondria by binding to Hsp70 to prevent oxidative stress-induced cell death and maintain mitochondrial homeostasis. However, a recent study showed that Bcl-2-associated athanogene 5 interacts with DJ-1 and attenuates the DJ-1-mediated protection of mitochondria, probably by shifting the subcellular distribution of DJ-1 and affecting its dimerization (42).

Moreover, there are also some possible targets of DJ-1 existing in the mitochondria, in particular mitochondrial complex I (43). It has been revealed that DJ-1 directly interacts with complex I subunits NDUFA4 and ND1, and the binding is enhanced under oxidative stress. In addition, complex I activity was shown to be reduced in DJ-1-knockdown NIH3T3 and human embryonic kidney 293 cells (43). Based on these findings, we speculate that DJ-1 is an integral mitochondrial protein that plays a role in maintaining mitochondrial homeostasis, including the integrity and activity of complex I.

### DJ-1 as a Regulator of Antioxidant Gene Expression

DJ-1 also acts as an antioxidant by upregulating antioxidant gene expression, in particular through the transcription factor NF-E2 related factor-2 (Nrf2), a critical inducer of antioxidant-responsive element (ARE)-mediated expression. Previous studies have shown that DJ-1 plays a role in stabilizing Nrf2 by affecting association with its repressor Kelch-like ECH-associated protein 1 (Keap1), and the subsequent ubiquitination of Nrf2 (44). The results of a recent finding supported the fact that DJ-1 promotes Nrf2–Keap1 dissociation, accounting for enhanced levels of nuclear translocation and ARE binding of Nrf2 in H9c2 cells when exposed to oxidative stress (44). In addition, an *in vivo* mouse kidney study reported that Nrf2 ubiquitination was increased in DJ-1 knockout (KO) mice compared with wild-type (WT) littermates (45). DJ-1 is responsible for the expression of several Nrf2 target genes including antioxidant enzyme NAD(P)H quinone oxidoreductase 1 and heme oxygenase-1, and products that contribute to the redox reaction such as thioredoxin 1 (Trx1) and glutathione (44–47).

**Figure 1:**
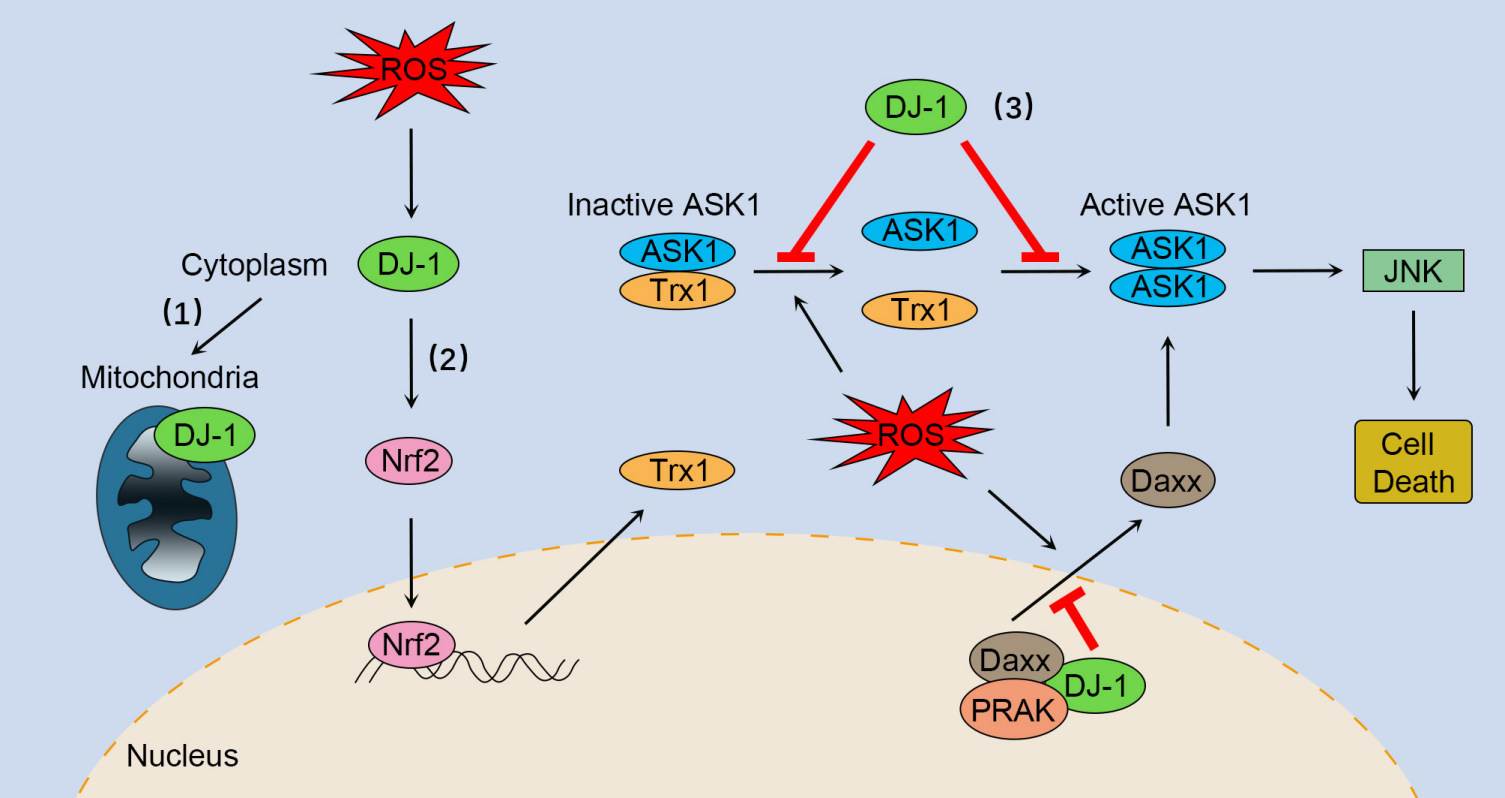
The multifaceted roles of DJ-1 as an antioxidant under oxidative stress. (1) DJ-1 translocates to the mitochondria and maintains mitochondrial homeostasis. (2) DJ-1 exerts antioxidant function by upregulating Nrf2 activation. (3) DJ-1 prevents oxidative stress-induced ASK1 signaling pathways by several mechanisms, including stabilizing the Trx1–ASK1 complex, increasing the expression of Trx1, disrupting ASK1 homodimerization through direct interaction, as well as sequestering the ASK1 activator Daxx in the nucleus. Nrf2, NF-E2 related factor-2; ASK1, apoptosis signal-regulating kinase 1; Trx1, thioredoxin 1; Daxx, death-associated protein 6; PRAK, p38-regulated/activated kinase; JNK, c-Jun N-terminal kinase.

However, some studies have indicated that activation of the Nrf2-ARE pathway is independent of DJ-1, and therefore it has been suggested that Nrf2 is a downstream effector of DJ-1 function (48). DJ-1 stimulates nuclear translocation of Nrf2 and enhances its recruitment to the Trx1 promoter (47). A study showed that DJ-1 had no influence on the binding between Nrf2 and Keap1 and ubiquitination state of Nrf2, indicating that DJ-1 functions through a non-Keap1-dependent mechanism (47). Consistent with this notion, downregulation of DJ-1 did not impair the Nrf2–Keap1 association in normal human corneal endothelial cells (49). Taken together, DJ-1 can exert antioxidant function by upregulating Nrf2 activation (Figure 1); however, the precise mechanisms need further research.

### DJ-1 as a Regulator of Oxidative Stress-Induced Apoptosis

DJ-1 plays a key role in regulating oxidative stress-induced apoptosis, especially preventing ASK1 activation through multiple mechanisms. First, DJ-1 can stabilize the inhibitory complex of Trx1–ASK1 (Figure 1). Under normal conditions, ASK1 is bound by Trx1 equally with or without DJ-1 overexpression (30). However, upon oxidative stress, Trx1 releases ASK1, a process that is suppressed by overexpression of DJ-1 (30). DJ-1 KO mouse brain homogenates are more susceptible to H_2_O_2_-mediated dissociation of Trx1–ASK1 complex compared with WT brains (30). In addition, DJ-1 can upregulate Trx1 expression through the Nrf2 pathway (Figure 1), thus enhancing intracellular levels of Trx1 and repressing ASK1 activation (47). Second, DJ-1 may disrupt ASK1 homodimerization through physical interaction, leading to inhibition of the ASK1 signaling pathway (Figure 1) (50). Of interest, one study suggested that H_2_O_2_ did not significantly impair the cytoplasm colocalization of ASK1 and DJ-1, while other studies have found that oxidative stress and DJ-1 with oxidized C106 are required for DJ-1/ASK1 association (29, 50, 51). Third, DJ-1 binds to death-associated protein 6 (Daxx) in the nucleus, blocking its translocation to the cytoplasm where Daxx activated its effector kinase ASK1 and the resulting apoptosis (Figure 1) (52, 53). Moreover, p38-regulated/activated kinase may be the essential partner of DJ-1, which induces DJ-1 to sequester Daxx in the nucleus and modulates oxidative stress-induced ASK1 activation (Figure 1) (53, 54).

## Role of DJ-1 in the Pathophysiology of Immune and Inflammatory Diseases

### DJ-1 and Sepsis

Sepsis, defined as a life-threatening, multiorgan dysfunction caused by a dysregulated host response to infection, remains the most leading cause of morbidity and mortality in intensive care units worldwide (55, 56). It is recently clear that sepsis features concomitant occurrence of excessive inflammation and immune suppression (57). Earlier diagnosis based on routine microbiologic cultures has been proposed in the “Surviving Sepsis” guidelines, which is key to antimicrobial therapy and improved outcomes of clinically ill patients (58). However, many cases have occurred that fail to identify the infecting organism and the inflammatory reaction often sustains after treatment of the infection, which is related to tissue damage and organ failure due to lack of effective therapy and supportive care (57, 59).

Macrophages play a crucial part in host immune and inflammatory response during all phases of sepsis. After infection, the activation of macrophages is mediated by a Toll-like receptor (TLR) that recognizes pathogen-associated molecular patterns, including LPS of gram-negative bacteria. In the early stage of sepsis, macrophages undergo M1 polarization and maintain a homeostasis by eliminating pathogens or damaged tissues and producing pro-inflammatory mediators (60). However, if the infection persists, macrophages can polarize toward the M2 phenotype and the host may present a LPS-tolerant state, leading to severe immunosuppressive stage of sepsis with deleterious consequences (61).

Cellular redox status plays a complicated role in the host immune response and outcomes of sepsis (62). Although excess ROS causes oxidative stress and cell injury, appropriate levels of ROS can initiate various signal transduction cascades that contribute to the bactericidal ability of macrophages and regulation of inflammatory reaction in host response (63, 64). Previous studies have shown that ROS is involved in modulating LPS-induced TLR4 trafficking to lipid rafts and subsequent TLR4 activation, as well as downstream signaling pathways in macrophages, such as mitogen-activated protein kinases (MAPKs) and nuclear factor kappa B (NF-κB) pathways (65, 66). Other studies have suggested that NADPH oxidase (NOX)-dependent ROS generation plays a critical role in this process, as TLR4 signaling and pro-inflammatory cytokine production are promoted by NOX activator but are suppressed by a NOX inhibitor (65). In addition, TLR4/NF-κB activation is involved in M1 macrophage polarization while the downregulated NF-κB pathway is connected to M2 polarization (67, 68).

DJ-1 has versatile functions and is distinguished as an antioxidant by multiple mechanisms. However, DJ-1 facilitates NOX-dependent ROS production in early active macrophages through direct interaction with p47^phox^, a subunit of NOX, leading to pro-inflammatory cytokine release (69). Compared with WT mice, *DJ-1*^−^^/–^ mice present with blunt TLR signaling that consequently impairs the bactericidal ability of macrophages, along with decreased local inflammation, and significantly increases mortality in a sepsis model (69). In addition, macrophages with restored DJ-1 expression were shown to rescue ROS generation and enhance survival in LPS-induced sepsis (69), suggesting that DJ-1 has a protective role during sepsis by controlling macrophage activation.

Interestingly, a recent study revealed that DJ-1/p47^phox^ binding disrupted the stability of the NOX complex and promoted subunit gp91^phox^ ubiquitination, thus influencing the optimal ROS production for bacterial clearance and M1 differentiation of macrophages (12). *DJ-1*^−^^/–^ mice exhibited elevated levels of pro-inflammatory mediators and improved survival and organ function compared with WT mice, and adoptive transfer of *DJ-1*^−^^/–^ bone marrow-derived mononuclear cells rescued WT mice from septic mortality (12). Additionally, circulating DJ-1 levels were increased and correlated with severity of sepsis and organ dysfunction in patients (12). Based on these findings, DJ-1 seems to act as a potent antioxidant that impairs optimal ROS levels for bacterial killing of macrophages and clinical outcomes of sepsis.

**Figure 2:**
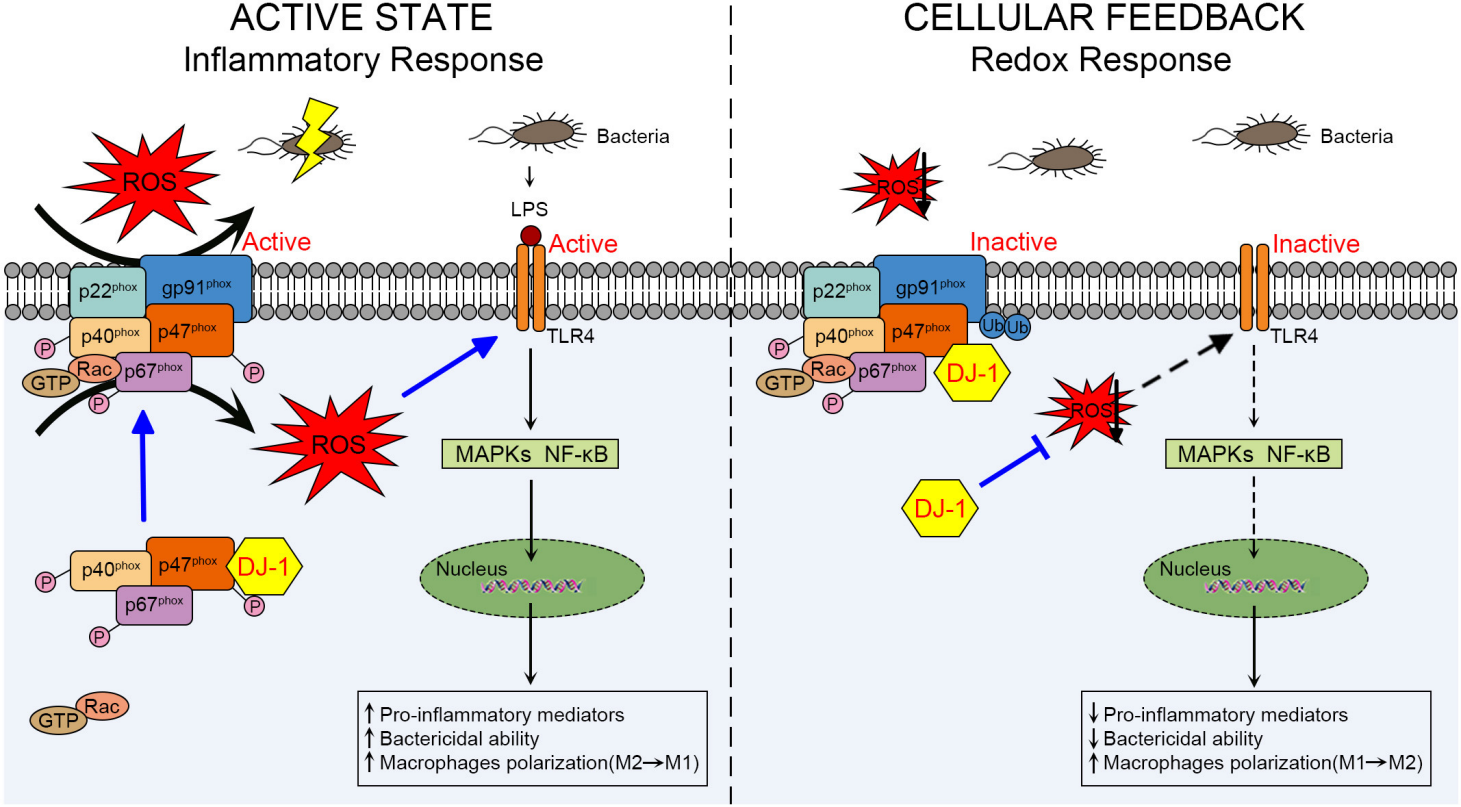
Schematic diagram of the role of DJ-1 in regulating macrophage activation through NOX-dependent ROS production and TLR4 signaling pathways during sepsis. In the active state of macrophages, DJ-1 probably exerts a protective function against LPS-induced sepsis. Through direct interaction with p47^phox^, DJ-1 promotes NOX-dependent ROS production, which facilitates TLR4/MAPKs and/or TLR4/NF-κB signaling pathways and results in releasing pro-inflammatory mediators, killing bacteria and polarizing to M1 phenotype. However, available DJ-1 functions as an antioxidant in cellular negative feedback to protect from oxidative stress and excessive inflammatory response. DJ-1 can reduce ROS at least through binding to p47^phox^, disrupting NOX stability and its ROS generating capacity, thereby impairing TLR4 activation and downstream signaling pathways. NOX, NADPH oxidase; TLR4, Toll-like receptor 4; LPS, lipopolysaccharide; MAPKs, mitogen-activated protein kinases.

It remains unclear how DJ-1 differs in the above two sepsis studies concerning its functional and molecular outcomes. In the active state of macrophages, DJ-1 probably exerts protective functions through NOX-dependent ROS production against sepsis to facilitate TLR4/MAPKs and/or TLR4/NF-κB signaling pathways, resulting in releasing pro-inflammatory mediators, killing bacteria, and polarizing to the M1 phenotype. However, available DJ-1 functions as an antioxidant in cellular negative feedback to protect from oxidative stress and excessive inflammatory response. DJ-1 can reduce ROS at least through binding to p47^phox^, disrupting NOX stability and its ROS generating capacity, thereby impairing TLR4 activation and downstream signaling pathways. A schematic diagram of our proposed mechanism is presented in Figure 2.

Since one study revealed that the C-terminus of DJ-1 is required for the DJ-1/p47^phox^ interaction (69), we consider that the different consequences of the physical association may be regulated or determined by the oxidation state of DJ-1, in particular C106 oxidation. In addition, we believe that the role of DJ-1 in sepsis is not limited to the DJ-1/p47^phox^/ROS axis. Rather, mitochondrial ROS are recognized as essential components of the innate immune response and bactericidal activity of macrophages (70).

Taken together, given the effects of DJ-1 on host immune defense by regulating ROS generation of macrophages, the pathophysiology of DJ-1 in sepsis remains to be revealed and the DJ-1/p47^phox^/ROS axis may become a potential therapeutic target to modulate the development of sepsis.

### DJ-1 and Allergic Diseases

IgE-mediated allergic diseases, such as asthma, rhinitis, and atopic dermatitis (AD), are mainly driven by MCs (71, 72). When the high-affinity IgE receptor FcεRI of MC surface is aggregated by multivalent antigen, the Src family tyrosine kinases are activated, in particular Lyn, leading to the recruitment and activation of spleen tyrosine kinase (Syk) and downstream signaling cascades, which ultimately causes the release of bioactive and inflammatory mediators (72, 73). In addition, other Src family kinases, such as Fyn, are also involved in antigen-induced MC activation (74).

ROS plays a role in modulating MC activation in both innate and acquired immune response, including allergic inflammatory reactions (75). Activated MCs promote the generation of ROS and inflammatory mediators through FcεRI cross-linking in response to antigen stimulation (76, 77). Diminished levels of DJ-1 and increased levels of ROS have been found in allergic patients with AD (14). Additional studies have shown that DJ-1 KO mice present with enhanced passive cutaneous anaphylaxis reactions and MC degranulation, compared with WT mice (14). In addition, lack of DJ-1 augments ROS generation and cytokine production in antigen-stimulated MCs (14). It has been suggested that DJ-1 deficiency causes excessive ROS levels in MCs, which differentially regulate the activation of Fyn and Syk (14). Therefore, DJ-1 modulates antigen-induced MC activation and allergic responses through ROS-dependent signal transduction cascades.

Of interest, a recent report showed that DJ-1 regulated human MC signaling by partially ROS-independent mechanisms (34). The study revealed that non-oxidized DJ-1 translocated and interacted directly with Lyn in lipid rafts after FcεRI engagement, initiating Lyn activation and downstream signaling pathways, but was only specific for human (34). Subsequently, cellular DJ-1 was oxidized along with the decline of ROS levels, thus preventing Syk deactivation to perpetuate MC signaling (34). Based on these findings, DJ-1 plays a unique dual role in FcεRI-activated human MC responsiveness, although the precise mechanisms are not completely clear. We speculate that lipid modification especially palmitoylation of three cysteine residues (C46/53/106) may be required for DJ-1 redistribution to lipid rafts and the C-terminal domain of DJ-1 may be required for interaction with Lyn.

Collectively, through ROS-dependent and/or ROS-independent mechanisms, DJ-1 is a vital regulator of MC-derived allergic disorders. In terms of an ROS-dependent mechanism, whether abnormal DJ-1/ROS levels influence other MC-involved immune and inflammatory response needs further investigation. Concerning the ROS-independent mechanism, the DJ-1/Lyn association proposes new therapeutic modalities for human allergic diseases or possibly other Lyn-mediated disorders.

### DJ-1 and AS

AS, a chronic inflammatory disease, is triggered by genetic susceptibility and environmental risk factors, which is the main pathological basis of ischemic cardio-cerebrovascular diseases, including coronary artery disease (78, 79). Accumulating evidence has demonstrated the essential role of T cells as drivers and modifiers in AS (80). Mass cytometry has characterized distinct CD4^+^ T cells that were activated and differentiated by T cells in human AS plaque tissue (81). After migration toward the activated endothelial layer, CD4^+^ T cells are critical for local antigen-presenting cells within early intimal fatty streaks, such as dendritic cells and macrophages and associated inflammation and AS progression (82, 83).

**Figure 3:**
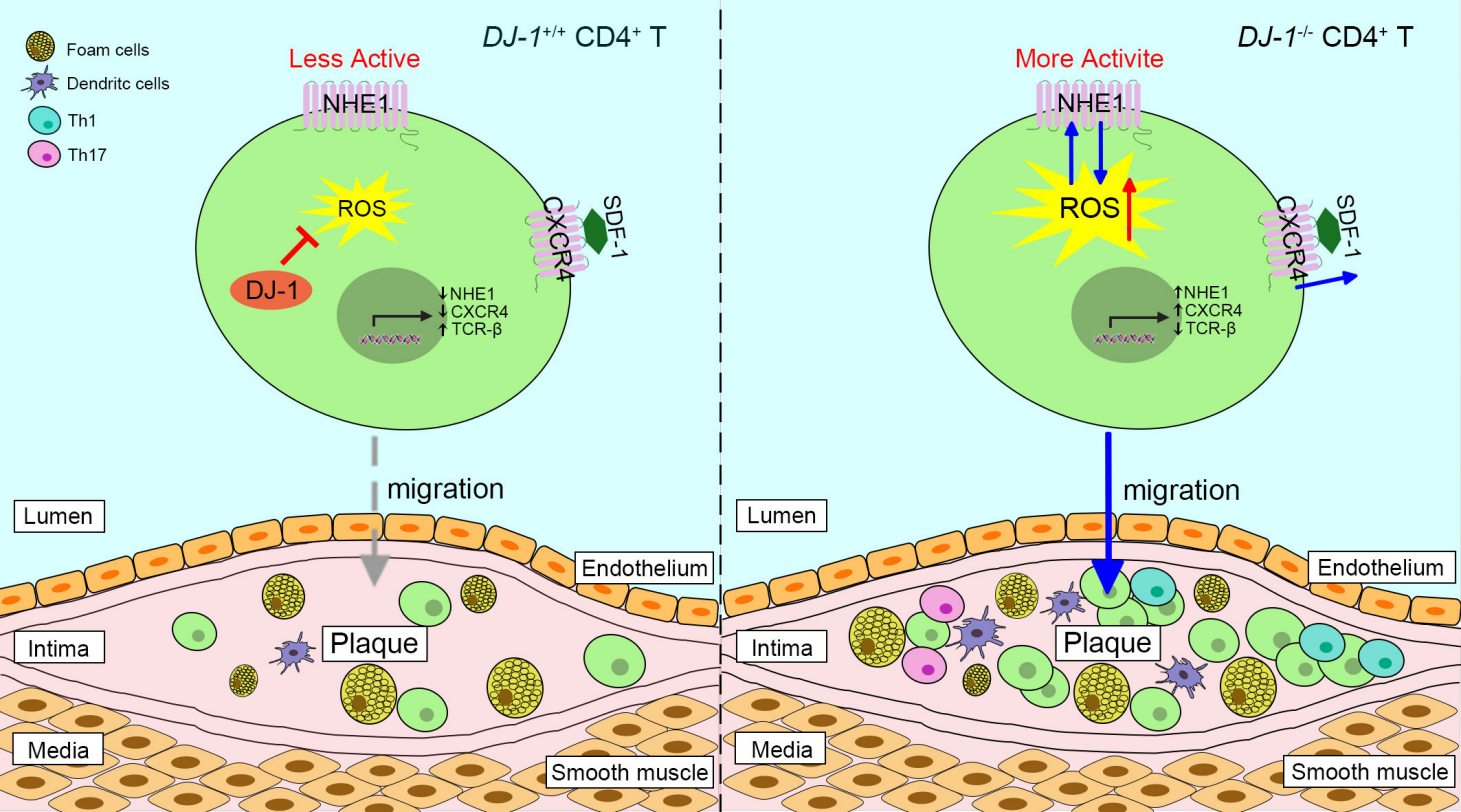
Proposed scheme of the role of DJ-1 in atherosclerosis plaques progression. Absence of DJ-1 leads to excess ROS, which could promote the expression and activity of NHE1 as well as the activity of CXCR4 in response to SDF-1, thus driving CD4^+^ T cell migration and infiltration into intima. In addition, DJ-1-deficient CD4^+^ T cells are prone to differentiate into pro-inflammatory Th1 and Th17 phenotypes. Therefore, lack of DJ-1 contributes to atherosclerosis plaque progression. NHE1, Na^+^/H^+^ exchanger 1; CXCR4, chemokine receptor 4; SDF-1, stromal cell-derived factor 1.

Studies have shown that *DJ-1*^−^^/–^ mice present with higher levels of neointimal plaque formation and increased accumulation of CD3^+^ T cells in the plaque formed by carotid artery ligation, compared with WT mice (13, 84). It is likely triggered by the elevated proliferation of DJ-1 deficient CD3^+^ T cells and enhanced migration in response to stromal cell-derived factor-1 via overexpression of chemokine receptor 4 (13, 85). In addition, a new relationship between the DJ-1-ROS-Na^+^/H^+^ exchanger 1 (NHE1) revealed that DJ-1-deficient CD4^+^ T cells upregulated the expression and activity of NHE1, which may have resulted from enhanced ROS generation (86). In turn, NHE1 activity can impair ROS production (86). In this respect, lack of DJ-1 promotes neointima formation since NHE1 contributes to cell migration (87). Of interest, DJ-1 may take a part in the redox regulation of T cell receptor (TCR) signaling. The expression of TCR signaling proteins such as CD3 and TCR-β is dramatically diminished in DJ-1-deficient activated T cells, while co-stimulatory CD28 is upregulated (86). Moreover, DJ-1-deficient CD4^+^ T cells show more potential to differentiate into pro-inflammatory Th1 and Th17 phenotypes in an AS model (Figure 3) (13). Taken together, it appears that DJ-1 is a negative regulator of CD4^+^ T cell migration and activation, thereby suppressing AS pathology.

### DJ-1 and MS

MS is a chronic autoimmune disease of the central nervous system with no cure currently, which is triggered by various environmental factors in genetically susceptible individuals (88, 89). Recent literature suggests that astrocytes play dual roles in the evolution of MS lesions. They not only adopt a neurotoxic phenotype and act as immunocompetent cells but also promote neuroprotection and axonal preservation (90). Emerging evidence has revealed that ROS has implications in the pathology of MS and experimental autoimmune encephalomyelitis (EAE), the most widely used animal model of MS, and contributes to demyelination and axonal damage of the central nervous system (91–93).

Previous studies have shown that the expression of DJ-1 is upregulated in the brains of EAE mice as well as the cerebrospinal fluid from relapsing–remitting MS patients, along with increased disease severity (94, 95). Active astrocytes express higher levels of DJ-1 and Nrf2 in both active and chronic inactive MS lesions, compared with control brain tissue and normal-appearing white matter (96). In addition, DJ-1-deficient astrocytes are prone to enhanced inflammation, including cytokine production and oxidative stress (97, 98). Based on these findings, we consider that DJ-1 may participate in astrocyte-mediated protective effects in MS that are at least partially dependent on Nrf2 signaling pathways. However, endogenous regulation is not sufficient to prevent ROS-induced cellular damage, which is abundant in inflammatory MS lesions. In this respect, molecules that target DJ-1/Nrf2 signaling may ameliorate MS development, since dimethyl fumarate that has been FDA-approved for the treatment of MS can upregulate Nrf2 in astrocytes.

## DJ-1 as a Secretory Protein

Previous studies have shown that DJ-1 is a secretory protein and can serve as a biomarker of cancer, stroke, and early phases of PD, which could be beneficial for diagnosis, monitoring, and prognosis (99–101). As mentioned above, plasma DJ-1 protein levels are increased and correlate with sepsis severity and poor clinical outcomes in patients (12). DJ-1 levels are upregulated in the cerebrospinal fluid from MS patients and may have implications with disease progression (95). In FcεRI-activated human MCs, total intracellular expression of DJ-1 does not change but DJ-1 with oxidized C106 is upregulated, coinciding with both DJ-1 secretion and diminished intracellular ROS levels (14). In addition, oxidized forms of DJ-1 are preferentially secreted through microdomains and the amounts of extracellularly secreted DJ-1 are only a fraction of the cellular DJ-1 content (34, 102). However, diminished levels of DJ-1 and increased levels of ROS are found in AD patients compared with healthy volunteers, in which alterations are not related to disease severity (14). To the best of our knowledge, DJ-1 can be secreted from various cells because of its ubiquitous expression, but the precise mechanisms remain unknown. Under stress conditions, DJ-1 acts as a sensor and plays a role in regulating cell responses. Damaged cells most likely upregulate the expression of DJ-1 and secrete it in order to attenuate environmental and intracellular oxidative stress at least by self-oxidation to maintain homeostasis (103). Therefore, it will be of clinical importance to determine if secretion of DJ-1 has any disease-specific role or function.

## Perspectives on DJ-1 as a Therapeutic Target of Immune and Inflammatory Diseases

Here, we reviewed the critical roles of DJ-1 in several immune and chronic inflammatory diseases. The high levels of DJ-1 could be a candidate biomarker for sepsis and MS, while the extent of DJ-1/ROS abnormality might be relevant to the onset of AD. Taking into consideration that the pivotal functions of DJ-1 involved with activation of macrophages, MCs and T cells, it is a promising therapeutic target for various immune and inflammatory diseases. Although there is no clinical drug targeting DJ-1 for immunotherapy, we can comment on potential treatment strategies for future directions.

As discussed above, DJ-1 can act as an antioxidant against oxidative stress and prevent the development of AS and MS. DJ-1-binding compounds, including UCP0045037/compound A, UCP0054278/compound B, and compound-23 identified from the university compound library and zinc compound library, prevent superfluous oxidation of DJ-1 and maintain reduced DJ-1, which inhibits oxidative stress-induced toxicity in *in vitro* and *in vivo* PD and stroke models (104, 105). Recently, a DJ-1-based peptide named ND-13 was found to protect cultured cells against oxidative insults through activating Nrf2 signaling and significantly improve outcomes in a mouse model of PD (106). In addition, some drugs or drug candidates have also been studied to test their capability of facilitating DJ-1 expression or activation. For example, sodium phenylbutyrate, a histone deacetylase inhibitor, is reportedly neuroprotective in several neurodegenerative disease animal models (107). Other studies have revealed that sodium phenylbutyrate upregulates DJ-1 expression in both cultured cells and mice brains, which is required for its broad protection from metabolic insults (107). Therefore, these compounds/molecules represent potential therapeutic modalities for AS and a wide range of neurodegenerative diseases, including MS, since they can cross the blood–brain barrier and exert protective effects against oxidative stress.

In macrophage-involved sepsis, the DJ-1/p47^phox^ interaction is a decisive factor in regulating ROS-dependent macrophage activation and LPS-responsiveness. It is of great significance to unveil the relative influence of cellular context that leads to different consequences of DJ-1/p47^phox^ interaction, as well as the crystal structure of the DJ-1/p47^phox^ complex. After solving these problems, the DJ-1/p47^phox^/ROS axis may become an effective therapeutic target for sepsis.

In human allergic diseases, DJ-1 was identified as a central regulator of MC activation in response to antigen stimulation (34). Allergic inflammatory responses such as degranulation, cytokines, and eicosanoid production of human MCs were inhibited after transfecting into DJ-1-targeted short hairpin RNAs, suggesting that transcriptional knockdown of DJ-1 using short hairpin RNAs may be an effective strategy to treat DJ-1-dependent human allergic disorders (34). In addition, further characterization of DJ-1/Lyn interaction may open novel avenues of therapeutic options in MC-mediated immune and inflammatory diseases.

## Author Contributions

QW conceived the review article and made the corrections in the manuscript. BY and QH provided some critical comments. LZ wrote the manuscript. JinW and JiaW collect the related research articles. All authors contributed to manuscript revision, read, and approved the submitted version.

## Conflict of Interest

The authors declare that the research was conducted in the absence of any commercial or financial relationships that could be construed as a potential conflict of interest.

## Abbreviations

AS: atherosclerosis
ASK1: apoptosis signal-regulating kinase 1
CXCR4: chemokine receptor 4
Daxx: death-associated protein 6
EAE: experimental autoimmune encephalomyelitis
H_2_O_2_: hydrogen peroxide
Hsp70: heat shock protein 70
Keap1: Kelch-like ECH-associated protein 1
KO: knockout
LPS: lipopolysaccharide
MAPKs: mitogen-activated protein kinases
MCs: mast cells
MS: multiple sclerosis
NF-κB: nuclear factor kappa B
NHE1: Na^+^/H^+^ exchanger 1
NOX: NADPH oxidase
Nrf2: NF-E2 related factor-2
PD: Parkinson's disease
ROS: reactive oxygen species
SDF: stromal cell-derived factor
SO_2_H: sulfinic acid
SO_3_H: sulfonic acid
SOH: sulfenic acid
Syk: spleen tyrosine kinase
TCR: T cell receptor
TLR: Toll-like receptor
Trx1: thioredoxin 1
WT: wild-type.
